# Manual and automated methods for identifying potentially preventable readmissions: a comparison in a large healthcare system

**DOI:** 10.1186/1472-6947-14-28

**Published:** 2014-04-05

**Authors:** Ana H Jackson, Emily Fireman, Paul Feigenbaum, Estee Neuwirth, Patricia Kipnis, Jim Bellows

**Affiliations:** 1 Care Management Institute, Kaiser Permanente, Oakland, California, USA; 2 The Permanente Medical Group, Oakland, California, USA; 3 Management Information and Analysis, Kaiser Permanente Northern California, Oakland, California, USA

**Keywords:** Qualitative research, Quality assurance, Health care/methods, Patient readmission/statistics & numerical data

## Abstract

**Background:**

Identification of potentially preventable readmissions is typically accomplished through manual review or automated classification. Little is known about the concordance of these methods.

**Methods:**

We manually reviewed 459 30-day, all-cause readmissions at 18 Kaiser Permanente Northern California hospitals, determining potential preventability through a four-step manual review process that included a chart review tool, interviews with patients, their families, and treating providers, and nurse reviewer and physician evaluation of findings and determination of preventability on a five-point scale. We reassessed the same readmissions with 3 M’s Potentially Preventable Readmission (PPR) software. We examined between-method agreement and the specificity and sensitivity of the PPR software using manual review as the reference.

**Results:**

Automated classification and manual review respectively identified 78% (358) and 47% (227) of readmissions as potentially preventable. Overall, the methods agreed about the preventability of 56% (258) of readmissions. Using manual review as the reference, the sensitivity of PPR was 85% and specificity was 28%.

**Conclusions:**

Concordance between methods was not high enough to replace manual review with automated classification as the primary method of identifying preventable 30-day, all-cause readmission for quality improvement purposes.

## Background

Hospital readmissions are expensive and may reflect poor quality care. Under the new Readmissions Reduction Program, the U.S. Centers for Medicare and Medicaid Services reduces payments to hospitals with excess 30-day readmission rates [1]. Many hospitals are therefore interested in identifying preventable readmissions and understanding how they can be prevented.

Classifying readmissions as potentially preventable or not preventable can be used to improve hospital performance. Administrators can sort potentially preventable readmissions into categories that are actionable for improvement. They can identify trends over time or across reporting units. Classifying readmissions as potentially preventable or not preventable can also be used to establish accountability across reporting units and reward top performers.

In a recent meta-analysis of 16 studies, the median proportion of 30-day readmissions that were judged as avoidable was 21.6% [2]. The range was 5% to 59% [2-4]. The methods used to measure potential preventability vary greatly, but most involve manual chart review by at least one reviewer [2,5]. Manual review is labor intensive and subjective. To address these shortcomings, automated software classification programs have been developed that rely on administrative data to identify potential preventability [5,6]. Automated classification offers the prospect of greater efficiency and consistency. However, automated classification has been found to identify more readmissions as potentially preventable than does manual review, so its validity has been questioned [5,7]. Although studies have compared manual review to automated classification, no published evidence describes the extent of agreement between methods applied to the same readmissions. We assessed the concordance between manual review and automated classification on the same set of readmissions to determine if automated classification could more efficiently identify preventable readmissions for quality improvement purposes.

## Methods

### Design

We compared a manual review of readmissions to automated classification by the Potentially Preventable Readmission (PPR) software from 3 M. Manual review consisted of a multi-step process that has been described in more detail elsewhere, which was conducted to identify missed opportunities to prevent readmissions [8]. The first step was a detailed chart review conducted by trained nurse reviewers, based loosely on an expanded version of a readmissions diagnostic tool from the Institute for Healthcare Improvement [9]. Chart review data came from KP HealthConnectTM, the electronic health record (EHR). Interviews with treating physicians followed, and guided topics included their assessment of the preventability of readmission. We also interviewed patients and family caregivers in 73% of readmissions, again using an interview guide and soliciting an assessment of preventability. The same nurse reviewer conducted the chart review and interviews for each patient. In the final step of manual review, the nurse reviewer partnered with a physician reviewer to review and assess information and opinions from the chart review and interviews. They identified factors representing missed opportunities to prevent the readmission from a checklist of 35 possibilities prepared by subject matter experts and pilot tested before use. After discussion, the nurse reviewer/physician team used clinical judgment to assess the preventability of the readmission on a five-point scale (not, slightly, moderately, very, or completely likely to be preventable).

Preventability was assessed within six weeks of readmission. Nurse reviewers conducting chart reviews and interviews were trained and, during each case review, received facilitation, guidance, and consultation from a member of the research team that focused on data validation and consistent ratings during data collection.

The PPR software assesses potential preventability based on All Patient Refined Diagnosis Related Groups (APR DRGs), an index of diagnosis and severity of illness [10]. Cases are assigned an APR DRG code at initial admission and at readmission. A panel of physicians involved in the development of PPR looked at all possible combinations of APR DRGs and predetermined whether each combination involved a readmission that was clinically related to initial admission or the result of a complication from initial admission^7^. If an APR DRG combination was predetermined to be clinically related or involving a clinical complication, PPR classifies the readmission as potentially preventable [11].

### Setting and population

Our study was conducted in the Northern California region of Kaiser Permanente (KPNC) which currently has 3.25 million members and had 20 hospitals at the time of this study. We piloted and refined our manual review methods at two hospitals. The readmissions we reviewed for this assessment took place between December 2009 and June 2010 in the remaining 18 KPNC hospitals; at the time, the regional all cause, 30-day readmission rate for Medicare recipients was 12.2%, well below the 19.6% reported across all fee-for-service Medicare beneficiaries in the United States (unadjusted for any case mix differences) [12,13]. The hospitals were located in both urban and suburban areas, and the daily census ranged from 35 to 250 patients. All hospitals employed salaried hospitalists, and five were teaching hospitals.

Our comparison included 459 cases of patients readmitted for any reason within 30 days of hospital discharge for whom manual reviews had been conducted as part of a previous quality improvement report [8]. The initial population, identified using administrative data from the EHR, consisted of approximately 30 patients at each site who were most recently and consecutively readmitted to the same facility within 30 days of index hospitalization discharge. We chose the most recent readmissions to increase the likelihood of reaching patients, families, and providers for interviews and ensuring they would recall the details of the episode. We excluded patients who were pregnant, childbearing, or under the age of 18 from the initial population. In addition, during the assessment reported here, we excluded 79 readmissions because the manual review was missing data (17) or did not assess readmission preventability (62), and the PPR software excluded 71 readmissions due to diagnoses of human immunodeficiency virus (HIV) or metastatic malignancy (64), patients who left against medical advice (2), transfers to other settings (4), or multiple trauma (1). Nine readmissions were both manual review and PPR exclusions. Each of the remaining 459 readmissions, which had an existing assessment of preventability from manual review was independently classified as preventable or not preventable by automated classification using PPR.

### Analysis

We compared results of manual review and PPR using two-by-two tables to describe patterns of agreement and disagreement. The true proportion of readmissions that are potentially preventable is unknown, but the predominant reported method of identifying potentially preventable readmissions is manual review [5]. We therefore used manual review as a reference point to measure the sensitivity and specificity of PPR. Sensitivity refers to the percentage of potentially preventable readmissions identified by manual review that were also identified as such by PPR. Specificity refers to the percentage of non-potentially preventable readmissions identified by manual review that were also identified as such by the software program.

We conducted two supplementary analyses, examining PPR sensitivity and specificity among medical patients and among readmissions occurring within and after seven days of hospital discharge. The Kaiser Permanente Northern California Institutional Review Board approved this study.

## Results

The median age of readmitted patients was 69; 54% were female (Table 1). PPR identified 78% (358) of readmissions as potentially preventable, whereas the manual review identified 49% (227) of readmissions as potentially (slightly to completely) preventable. Overall, the methods agreed about preventability or non-preventability in 56% (258) of cases (Table 2).

**Table 1:** Patient characteristics (n = 459)

	
Mean age, years	68.7
Female, %	54
Preference for English language, %	94
Race/ethnicity, %	
White	66
African American	11
Asian	9
Latino	9
Mean length of stay, days	6.4
Mean ED visits in prior 6 months	1.13
Mean inpatient admissions in prior 6 months	1.07
Service type, %	
Medical	81.2
Surgical	17
Functional status, %	
Fully or partially dependent	60
Independent	40

**Table 2:** Concordance between methods for identifying potential preventability

		**Manual review**		**Total**
		**Potentially preventable**	**Not potentially preventable**	
		**N = 227**	**N = 232**	
PPR	Potentially preventable N = 358	192	166	258
	Not potentially preventable N = 101	35	66	101
Total		227	232	459

Using manual review as reference, the sensitivity of PPR was 85%. In other words, it identified 85% of the potentially preventable readmissions that were identified by manual review. The specificity of PPR was 28%; it correctly classified 28% of the non-potentially preventable readmissions identified by manual review. Of the 232 cases identified as not potentially preventable by manual review, PPR identified 72% as potentially preventable. These results did not vary substantially when we considered only medical patients (N = 312). When we considered readmissions that occurred within seven days of discharge from the index admission (N = 326), sensitivity was slightly higher at 91%; however, when we considered 133 readmissions that occurred more than seven days after index discharge (N = 133), sensitivity was 67%. We did not test the statistical significance of this difference. Specificity did not vary substantially from the original analysis.

## Discussion

Substantial differences existed between manual review and automated classification methods, with PPR identifying many more readmissions as potentially preventable. This may have occurred because PPR uses a sole criterion to identify potential preventability: clinical relatedness to the index admission. In contrast, manual review classified as non-preventable many readmissions that were clinically related to the index stay. For example, a 75-year-old man was admitted twice within 30 days for exacerbation of chronic obstructive pulmonary disease. Reviewers found that his follow-up care and transition care plan were appropriate. The patient and his physician felt that the readmission could not have been prevented by Kaiser Permanente, and the reviewers agreed.

To a lesser extent, manual review also identified potentially preventable readmissions that PPR did not identify. For example, a 54-year-old woman was first admitted for partial thickness burns and then readmitted with a digestive system diagnosis. Reviewers found that, had she received appropriate referrals and post-discharge follow up, the readmission may potentially have been prevented. This assessment is consistent with recent research suggesting that, immediately after discharge, patients may be at generalized elevated risk and need additional support to manage ongoing health conditions [14]. PPR did not identify this case as potentially preventable.

Manual review, a subjective process, might have resulted in misclassifications. Manual review processes including more than one reviewer are associated with an increase in the proportion of readmissions identified as preventable [2]. Our manual review process used a nurse reviewer/physician team to assess preventability and identified 47% of readmissions as potentially preventable, nearly double the reported median [2]. It is unlikely that between-methods differences resulted from underestimation of preventability on manual review.

A strength of our report is that we used both methods among the same cases, controlling for variables that have made it difficult to compare methods of measuring preventability in the past, such as patient population and quality of hospital care [15]. Several limitations deserve mention. Reviewers were affiliated with (physicians) or employed by (nurses) Kaiser Permanente, which might have affected their assessment; however, they had not provided care for cases they reviewed. Our assessment took place in an integrated care setting with comprehensive EHR capabilities, and the generalizability of our findings to other settings is unknown. PPR is designed to assess potential preventability over thousands of cases; our analysis may have been too small to assess its accuracy. A different automated classification system may have generated different results, although studies using administrative data alone yield preventability estimates of 55% to 77.1%, much higher than the median for manual review of less than 22% [2].

Few validation reports of PPR exist to which we can compare our results. PPR identified 6.2% of 30-day readmissions among pediatric patients as potentially preventable and excluded some diagnoses amenable to quality improvement or of uncertain preventability; the authors concluded that caution was warranted when applying the tool to pediatric populations [16]. In preliminary findings from an ongoing study at the U.S. Department of Veterans Affairs (VA), PPR identified just over half of researcher-identified pneumonia readmissions [17]. Another VA study found that PPR and the Centers for Medicare & Medicaid Services (CMS) all-cause readmission measure were moderately correlated; when the variable of potential preventability was removed from the analysis, correlation increased [18].

Our objective was to determine whether PPR could replace manual review as a method for identifying preventable readmissions to support our ultimate goal of identifying system gaps that contributed to them. The significant discrepancy between results precludes that option; PPR classification agreed with manual review only slightly better than half the time. It would overlook 15% of preventable readmissions and direct most of our organizational attention to readmissions that were not potentially preventable.

The developers of PPR recommend that it be used as a screening tool to identify types of patients and providers with higher than expected readmission rates as a means of focusing subsequent manual review on those patients who have the greatest likelihood of having a preventable readmission. We did not assess the use of PPR across settings and cannot comment on its ability to measure relative performance across facilities. However, in a recent comparison of PPR and the CMS all-cause readmission measure, PPR hospital profiles would have generated different payment penalties for 30% of hospitals [18].

The true number of potentially preventable readmissions remains unknown, and the choice of method greatly influences the proportion identified as potentially preventable. However, meaningful identification of preventability, which pinpoints missed opportunities leading to avoidable readmissions and forms the basis for quality improvement efforts, depends on the review of primary data [2,8,18]. Future research is required to identify and test ways to refine the PPR to increase its concordance with manual review. For example, studies with larger samples may identify subsets of readmissions in which sensitivity and specificity are improved. For instance, one of our additional analyses suggests that PPR sensitivity may vary with the timing of readmissions; further research is required to confirm this finding. Research is also required to establish the effectiveness of using automated classification and manual review in combination to identify potentially preventable readmissions and quality improvement opportunities to address them.

## Conclusions

Thorough manual review and automated classification methods differed substantially in the proportion of readmissions classified as potentially preventable. PPR identified many more readmissions as potentially preventable. Not enough concordance currently exists between methods to use automated classification to replace manual review for quality improvement initiatives.

## Competing interests

All authors declare that they have no competing interests.

## Authors’ contributions

AJ designed the study, collected, analyzed and interpreted the data, and revised the manuscript for important intellectual content. EF collected and analyzed data and drafted the manuscript. PF interpreted the data and revised the manuscript for important intellectual content. EN designed the study, collected data, and revised the manuscript. PK analyzed the data and revised the manuscript. JB conceived of the study, interpreted the data, and revised the manuscript. All authors approved the final version of the manuscript.

## Pre-publication history

The pre-publication history for this paper can be accessed here:

http://www.biomedcentral.com/1472-6947/14/28/prepub
